# Effect of Food Residues in Biofilm Formation on Stainless Steel and Polystyrene Surfaces by *Salmonella enterica* Strains Isolated from Poultry Houses

**DOI:** 10.3390/foods6120106

**Published:** 2017-11-29

**Authors:** Alba María Paz-Méndez, Alexandre Lamas, Beatriz Vázquez, José Manuel Miranda, Alberto Cepeda, Carlos Manuel Franco

**Affiliations:** Laboratorio de Higiene, Inspección y Control de Alimentos, Dpto. de Química Analítica, Nutrición y Bromatología, Universidad de Santiago de Compostela, 27002 Lugo, Spain; albamaria.paz@rai.usc.es (A.M.P.-M.); beatriz.vazquez@usc.es (B.V.); josemanuel.miranda@usc.es (J.M.M.); alberto.cepeda@usc.es (A.C.); carlos.franco@usc.es (C.M.F.)

**Keywords:** *Salmonella*, biofilm, morpothypes, stainless steel, food residues, tomato, poultry, milk

## Abstract

*Salmonella* spp. is a major food-borne pathogen around the world. The ability of *Salmonella* to produce biofilm is one of the main obstacles in reducing the prevalence of these bacteria in the food chain. Most of *Salmonella* biofilm studies found in the literature used laboratory growth media. However, in the food chain, food residues are the principal source of nutrients of *Salmonella*. In this study, the biofilm formation, morphotype, and motility of 13 *Salmonella* strains belonging to three different subspecies and isolated from poultry houses was evaluated. To simulate food chain conditions, four different growth media (Tryptic Soy Broth at 1/20 dilution, milk at 1/20 dilution, tomato juice, and chicken meat juice), two different surfaces (stainless steel and polystyrene) and two temperatures (6 °C and 22 °C) were used to evaluate the biofilm formation. The morphotype, motility, and biofilm formation of *Salmonella* was temperature-dependent. Biofilm formation was significantly higher with 1/20 Tryptic Soy Broth in all the surfaces and temperatures tested, in comparison with the other growth media. The laboratory growth medium 1/20 Tryptic Soy Broth enhanced biofilm formation in *Salmonella*. This could explain the great differences in biofilm formation found between this growth medium and food residues. However, *Salmonella* strains were able to produce biofilm on the presence of food residues in all the conditions tested. Therefore, the *Salmonella* strain can use food residues to produce biofilm on common surfaces of the food chain. More studies combining more strains and food residues are necessary to fully understand the mechanism used by *Salmonella* to produce biofilm on the presence of these sources of nutrients.

## 1. Introduction

*Salmonella* spp. are major food-borne pathogens around the world. The *Salmonella* genus is composed by two species, *S. bongori* and *S. enterica*. Also, the latter is also composed of six subspecies: *S. enterica* (I), *S. salamae* (II), *S. arizonae* (IIIa), *S. diarizonae* (IIIb), *S. houtenae* (IV), and *S. indica* (VI) [1]. In the year 2015, *S. enterica* was responsible of 94,625 confirmed cases of salmonellosis and 126 deaths in the European Union (EU). Although in the last decade the cases of human salmonellosis followed a negative trend, the last report of the European Food Safety Agency (EFSA) showed a slight increase in the number of infections [2]. These results reveal the importance of continuing developing new strategies to avoid the persistence of *Salmonella* strains through the food supply chain. For this purpose, it is of great importance to fully understand the survival mechanism of this pathogen in the different environments of the food chain. The sources of *Salmonella* in the food chain are mainly poultry products such as chicken meat and eggs [2]. However, in the last years, fresh products such as vegetables have been also responsible of salmonellosis outbreaks due to, among other things, the use of polluted irrigation water. The presence of *Salmonella* strains in fresh products is a major public health problem as preservatives are not commonly used in these products and they are normally consumed raw [3,4].

One of the most important persistence mechanisms of *Salmonella* is biofilm formation. A biofilm is defined as a community of microorganisms of the same or different species enclosed in a self-produced polymeric matrix adhered to different kinds of live or abiotic surfaces [5]. Biofilms cells are characterized by an increased resistance to environmental stresses (i.e., UV radiation, pH change, osmotic shock, and desiccation), antimicrobials, biocides, and the host immune system in comparison with planktonic cells. Extracellular polymeric substances (EPSs) are one of the factors responsible of this protective effect [6]. The main EPSs in *Salmonella* biofilms are cellulose and curli fimbriae, whose combined production is the responsible of the RDAR (red, dry, and rough) morphotype. Although the relation between *Salmonella* virulence and RDAR morphotype is still unclear, it is demonstrated that *Salmonella* strains showing RDAR morphotype have a great ability to produce biofilm on abiotic surfaces [5,7]. The production of cellulose and curli fimbriae is closely related to the *csgD* and *adrA* genes. In the first place, the transcription of *csgD* results in the synthesis of the biofilm master regulator CsgD that directly activates the curli fimbriae biosynthesis genes and positively regulates the production of AdrA that activate the synthesis of cellulose through the *bcsA* gene [5]. *S. enterica* biofilm formation has been studied in a wide range of strains from different sources and under multiple environmental conditions being biofilm formation strain-dependent [8,9]. In addition, temperatures, nutrients, or oxygen levels highly influenced the amount of biofilm formed in *Salmonella* strains and the morphotype produced [10,11]. Therefore, the transcription of biofilm-related genes in *S. enterica* is closely related to the environmental conditions [12,13]. However, most of the studies carried out until now used laboratory growth media for biofilm formation studies. The results obtained in that kind of studies are only approximate because lab media have a well-balanced nutritional composition and do not represent the complex composition of the food products found in the food chain. For example, a recent study observed that growth media supplemented with meat juices residues increased biofilm formation in *S.* Typhimurium. Therefore, meat juices residues may act as a surface conditioner to support initial attachment to abiotic surfaces [14].

In this context, the aim of this study is to evaluate how food residues can influence the biofilm-forming ability of *S. enterica* strains belonging to three different subspecies and isolated from poultry houses. A common growth laboratory medium was used as a reference media. Tomato juice (vegetable industry), chicken juice (meat industry), and milk (dairy industry) were used as representations of the different products that can be processed in the food industry. In addition, to represent the different conditions that the strains can find in the different steps of the food chain, two surfaces (polystyrene and stainless steel) were tested in biofilm assays. Also, the morphotype and motility of all the strains in all the temperatures tested were determined.

## 2. Materials and Methods

### 2.1. Bacterial Strains and Growth Media

A total of 13 *Salmonella* strains belonging to three different subspecies of *S. enterica* were used in this study (Table 1). *Salmonella* strains were isolated from samples recollected from poultry houses as previously described [15]. The Kauffman–Whyte typing scheme for the detection of somatic (O) and flagellar (H) antigens, with standard antisera (Bio-Rad Laboratories, Hercules, CA, USA) was used to serotype *Salmonella* strains. *Salmonella* stock cultures were maintained at −20 °C in cryovials (Deltalab, Barcelona, Spain). These strains were revitalized by transferring one bead into 10 mL of Trypic Soy Broth (TSB, Oxoid, UK) and incubating for 24 h at 37 °C (precultures). To obtain the working cultures, 20 µL of *Salmonella* strains precultures were transferred into 10 mL of TSB and incubated 24 h at 37 °C. Four different growth media were used for biofilm assays. TSB at 1/20 (*w*/*w*) was used as a growth laboratory reference media. The nutrient balance of food residues found by *Salmonella* in the food chain is not as adequate as common laboratory growth media and therefore can have deficiency of some important components. In this sense, 1/20 TSB is a nutrient-limited medium that has demonstrated to be effective in promoting biofilm formation in *Salmonella* [8,16]. Due to this characteristic, 1/20 TSB was the growth medium chosen for comparative purposes. To represent possible food residues found in the food processing industry, tomato and chicken meat juice and UHT milk diluted 1/20 (*w*/*w*) were used in the biofilm assays. These assays were carried out at two different temperatures (6 °C and 22 °C) and two different surfaces (polystyrene and stainless steel).

### 2.2. Tomato and Chicken Meat Juice Preparation

Chicken meat juice (CMJ) was obtained as previously described by Birk et al. [17]. Briefly, chicken was obtained from local supermarkets and frozen for 2 days at −20 °C. Then, chicken was placed in a plastic bucket and thawed overnight. Chicken juice was collected in microtubes of 1.5 mL and centrifuged at 10,000× *g* for 10 min to eliminate large particles. The supernatant was filtered using 0.45 µm filter and stored at −20 °C until use. To obtain tomato juice (TJ), 50 g of tomato was mixed with 50 mL of distilled water in a bag and homogenized for 2 min. The liquid obtained was transferred to 1.5 mL microtubes and centrifuged at 10,000× *g* for ten minutes and the supernatant was filtered with 0.45 µm filters and stored at −20 °C until use.

### 2.3. Polystirene Biofilm Formation Assays

The determination of the biofilm formation in polystyrene with the different growth media was measured at 6 °C and 22 °C. Assays were carried out according Stepanovic et al. [16] with some modifications. Briefly, 96-well polystyrene microplates were filled with 200 µL of growth medium, and 20 µL of *Salmonella* culture containing 10^8^ CFU/mL after 24 h of incubation was added to each well. Then, the microplates were incubated under the tested temperatures for 48 h. After incubation, the liquid of the plate was poured off and the wells were washed three times with 300 µL of distilled water. *Salmonella* cells attached to the microplate walls were fixed using 250 µL of absolute methanol for 15 min and then the plates were emptied and air-dried. The wells were stained with 250 µL for 5 min with 0.1% crystal violet solution. Crystal violet was rinsed off by placing the microplate under running water. The microplates were air-dried, and the dye bound to the adherent bacterial cells was resolubilized using 250 µL of 33% glacial acetic acid. The optical density (OD) was measured at 630 nm with a Plate Reader (das, Roma, Italy). The assays were performed in triplicate in three independent experiments.

### 2.4. Stainless Steel Biofilm Formation Assays

Stainless steel coupons (3.5 × 3.5 cm) were used to determine biofilm formation of *Salmonella* strains with the different media and temperature tested in this study. The method used was an adaption based on Stepanovic et al. [16]. Briefly, the stainless steel coupons were placed at the bottom of 125 mL bottles (Deltalab, Spain) filled with 10 mL of the appropriate medium and 100 µL of overnight *Salmonella* culture containing 10^8^ CFU/mL. These bottles were incubated under the tested temperatures for 48 h. To remove non-adhered cells, the stainless steel coupons were washed with 10 mL of running distilled water, using a 10 mL micropipette. *Salmonella* attached to the stainless steel were fixed by immersing the coupons in absolute methanol for 15 min. After that, the coupons were air-dried and immersed in a 0.1% crystal violet solution for 5 min. The excess crystal violet was rinsed off by placing the stainless steel coupons under running water and air-drying. Finally, the coupons were placed in petri dishes containing 10 mL of 33% acetic acid to resolubilize the crystal violet. Finally, 200 µL of these solutions was poured in a 96-well microplate and the OD was measured at 630 nm with a plate reader (das, Roma, Italy).

### 2.5. Determination of Morphotype

The morphotype of the strains was determined at 6 °C and 22 °C as previously described by RömLing et al. [18] with some modifications. Briefly, TSB overnight *Salmonella* cultures were spread-plated onto Luria-Bertani (LB) plates without salt and supplemented with 40 mg/L of Congo red and 20 mg/L of Coomassie brilliant blue. The plates were incubated for 96 h and the morphotypes were determined in each strain at each temperature. The morphotypes in Congo red agar were classified as RDAR (red, dry, and rough; produce curli fimbriae and cellulose), SAW (soft and white), and SACW (soft and completely white; produce neither curli fimbriae nor cellulose and colonies were totally white).

### 2.6. Motility Assays

The motility of each strain in the different atmospheres was tested using a semisolid motility test medium according Karatzas et al. [19] with some modifications. The medium was composed by 10 g/L tryptone (Cultimed, Panreac, Barcelona, Spain), 5 g/L NaCl (Panreac, Barcelona, Spain), 4 g/L agar (Liofilchem, Roseto degli Abruzzi, Italy), 3 g/L beef extract (Oxoid Ltd., Thermo Scientific, Hampshire, UK), and 0.05 g/L of 2,3,5 triphenyltetrazolium chloride (Sigma-Aldrich, Taufkirchen, Germany), and was sterilized (15 min at 121 °C). Overnight cultures in TSB were transferred to the motility agar by stabbing. The plates were incubated at 22 °C and 6 °C for 72 h. *Salmonella* metabolism produces a red color when swimming away in the motility agar due to the reduction of 2,3,5 triphenyltetrazolium chloride to formazan. Finally, the ratio between the inoculum site and the edge of the red circle was measured as an indication of the motility.

### 2.7. Statistical Anaylisis

Statistical analyses were carried out with SPSS software for Windows (SPSS Inc., Chicago, IL, USA). Analysis of variance (ANOVA) was used to study the influence of growth media and temperature of incubation in the biofilm formation ability of *Salmonella* strains.

## 3. Results

**Figure 1:**
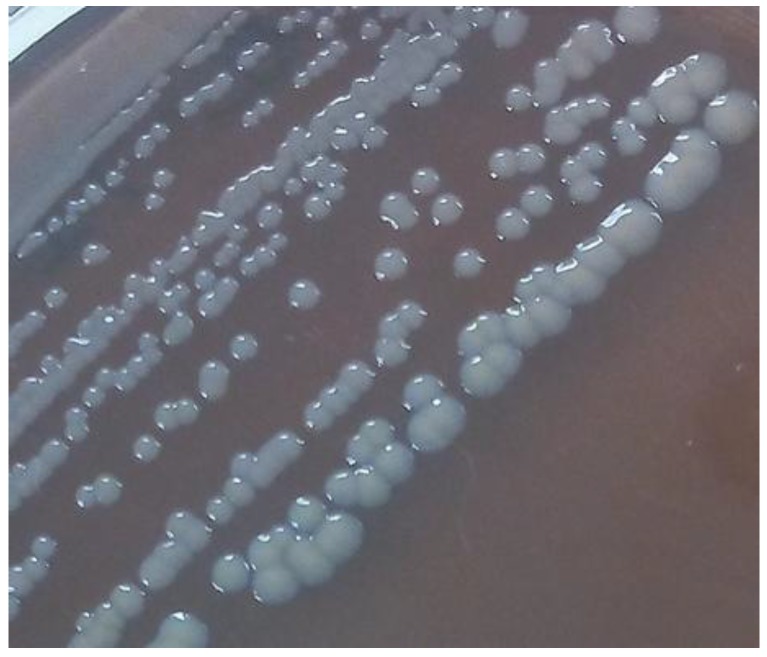
Morphotype Soft and Completely White (SCAW) produced by *Salmonella* strains at 6 °C.

A total of 13 strains isolated from poultry houses were used in this study (Table 1). The morphotype of *Salmonella* strains was evaluated under two different temperatures (6 °C and 22 °C). The results showed that *Salmonella* strains produced different morphotypes at different temperatures of incubation. All the strains produced the RDAR morphotype at 22 °C with the exception of *S. enterica* subsp. *arizonae* strains Lhica 2 and Lhica 6, which produced the SAW morphotype at 22 °C. However, at 6 °C, all the *Salmonella* strains tested in this study produced a morphotype characterized to be totally white, and therefore this morphotype was called by the authors as soft and completely white (SACW) (Figure 1).

The motility of *Salmonella* strains is closely correlated with their ability to produce biofilm. In this context, the motility of the strains used in this study was evaluated at 6 °C and 22 °C (Table 2). The mean motility of the strains was significantly higher (*p* < 0.05) at 22 °C than at 6 °C. There were also significant differences between the strains. At 22 °C, the motilities of *S.* Typhimurium Lhica T5 (25.10 ± 2.10 mm) and *S.* Enteritidis (25.40 ± 3.20 mm) were significantly higher than the motilities of the other strains. *S.* Infantis I5 presented lower motility at 22 °C (12.00 ± 1.00 mm). However, strains *S.* Infantis Lhica I4 (6.00 ± 0.50 mm) and *S.* Typhimurium T4 (6.00 ± 2.00 mm) presented higher motility at 6 °C. It is remarkable that no motility was detected in *S.* Typhimurium T1 and *S.* Newport N5 at 6 °C.

Biofilm formation by *Salmonella* strains used in this study was evaluated under two different temperatures (6 °C and 22 °C), two different surfaces (polystyrene and stainless steel), and four different growth media (1/20 TSB, 1/20 Milk, Tomato juice, and Chicken meat juice). Table 3 shows the mean OD_630_ values obtained for each strain in the biofilm assays in polystyrene at 6 °C and 22 °C. All the strains produced biofilm with 1/20 TSB, 1/20 milk, and CMJ in both temperatures. However, with TJ not all the strains produced biofilm. The cutoff value of 0.070 was established to consider biofilm formation by the strains tested. This cutoff value was calculated according the OD_630_ values obtained for the negative control wells (growth medium without strain) in polystyrene plates. Therefore, the strains that showed OD_630_ values lower than 0.070 in polystyrene assays were considered as not biofilm formed in those conditions. It is interesting that at 6 °C only one strain (*S.* Typhimurium Lhica T6) did not produce biofilm on TJ, and at 22 °C a total of five strains did not produce biofilm on TJ. In both temperatures, the mean OD_630_ average was significantly higher (*p* < 0.05) with 1/20 TSB than with the other growth media.

While there were no significant differences (*p* = 0.110) in the OD between 1/20 Milk, TJ, and CMJ at 6 °C, the growth medium 1/20 Milk showed significant higher (*p* < 0.05) OD values than the others two growth media at 22 °C. It is remarkable that both *S. enterica* subsp. *salamae* strains showed the higher OD_630_ values both in 1/20 TSB and 1/20 Milk media. It is especially interesting in the case of *S. enterica* subsp. *salamae* Lhica SA2 at 6 °C, which showed an OD_630_ value three times higher than *S.* Bardo Lhica B2, the non-*salamae* strain that showed the higher OD_630_ value.

The OD_630_ values obtained in stainless steel assays are not directly compared with those of polystyrene assays. Although the scientific principle is the same for both methods, they have slight differences in the quantities of reagents used. As in the case of polystyrene, the mean OD_630_ values were significantly higher (*p* < 0.05) with 1/20 TSB in both temperatures (6 °C and 22 °C). Between the other three growth media used, there were no significant differences (Table 4) at 6 °C and 22 °C, with the exception of TJ at 22 °C, where OD_630_ values were significantly lower (*p* < 0.05) than CMJ values.

While in polystyrene assays, *S. enterica* subsp. *salamae* strains presented the higher OD_630_ values in 1/20 TSB, in stainless steel assays the *S.* Enteritidis Lhica ET1 showed the higher OD_630_ values. The OD_630_ cutoff value established for stainless steel assays was 0.050. Therefore, all the strains were able to produce biofilm on stainless steel assays. All the strains presented higher OD_630_ values with 1/20 TSB, with the exception of *S.* Newport Lhica N5 at 6 °C, *S.* Typhimurium Lhica T6 at 22 °C, and *S. enterica* subsp. *arizonae* Lhica AZ6 at 6 °C and 22 °C, which presented higher values with CMJ. The incubation temperature also influenced the biofilm formation ability of the *Salmonella* strains tested in this study. In polystyrene and also in stainless steel, the mean OD_630_ values obtained with 1/20 TSB, 1/20 Milk, and CMJ were significantly higher at 22 °C than at 6 °C (Table 3 and Table 4). However, biofilm formation in TJ was not influenced by temperature in polystyrene and stainless steel.

## 4. Discussion

The morphotype produced by *Salmonella* strains is closely related with the ability to produce biofilm. In this sense, the *Salmonella* morphotype RDAR, characterized by the production of cellulose and curli fimbriae, is produced as a mechanism of resistance to environmental conditions [5]. Thus, it was observed that *Salmonella* turned off the genetic machinery related with the production of RDAR morphotype during in vivo infection and turned on this machinery when *Salmonella* was in the external environment again [20]. Most studies used temperatures of 28 °C or higher to evaluate the morphotypes produced by *Salmonella* strains. However, a study carried out by Lamas et al. [10] with *Salmonella* strains isolated from poultry observed that the morphotype produced by *Salmonella* strains varied with the different temperatures tested. While at 37 °C, most of the strains produced the SAW morphotype, at 20 °C most of the strains produced the RDAR morphotype. Nevertheless, it is also important to evaluate the effect of refrigeration temperatures in biofilm morphotype production. In this study, it was observed that, with the exception of *S. enterica* subsp. *arizonae* strains, all the strains produced the RDAR morphotype at 22 °C. By contrast, at 6 °C all the strains used in this study produced a morphotype not previously described in the literature to the best of our knowledge. This morphotype is characterized to present as totally white (Figure 1). Due to this characteristic, this morphotype was described by the authors as soft and completely white (SACW). A direct relationship between the morphotype produced by *Salmonella* strains at different temperatures and the biofilm formation in the different growth media cannot be established because morphotype was determined only in a specific medium (LB without NaCl and with Congo Red and Coomassie brilliant blue) for this determination. In addition to temperature, growth media can influence the morphotype produced by *Salmonella* strains. It is remarkable that the case of *S. enterica* subsp. *salamae* Lhica SA2 produced more biofilm on polystyrene at 6 °C than at 22 °C with 1/20 TSB. It is possible that the different food residues influence the production of cellulose and curli fimbria at refrigerated temperatures. On the other hand, the production of RDAR is not totally essential for the production of higher amounts of biofilm. For example, previous studies carried out by Seixas et al. [21] and Solomon et al. [8] found no differences in the amount of biofilm produced by RDAR and SAW morphotypes. It is possible that *Salmonella* strains at refrigerated temperatures activate other genetic mechanisms related with biofilm formation, as it could be the production of colanic acid or maybe the flagella that plays an essential role at these temperatures.

Motility mediated by flagella has an important role in *Salmonella* persistence and colonization. Also, it has been observed that motility contributes the internalization of *Salmonella* into host and plant cells [22,23]. The role of flagella in biofilm formation is not totally clarified, but flagella seems to be important for the initial attachment step to surfaces and not for biofilm maturation [5]. The results of this study showed that temperature highly influenced the biofilm formation. With the exception of TJ, biofilm formation was higher at 22 °C than at 6 °C in the growth media tested (Table 3 and Table 4). In the same way, the motility was significantly higher at 22 °C (Table 2). Low temperatures slow down the growth of microorganisms and may cause modifications in their metabolism that reduced their ability to produce biofilm. In this sense, it is possible that low temperatures reduced the synthesis of flagella in *Salmonella* cells, resulting in lower motility and lower capacity to attach to surfaces.

Previous studies [9,10,11,24] used growth laboratory media to perform biofilm assays. In the food industry, food residues are the principal source of nutrients used by food-borne pathogens. In an attempt to reproduce real conditions, this study used *Salmonella* strains isolated from the poultry industry to compare a common growth medium with different food residues on two common surfaces found in the food industry, polystyrene and stainless steel. For example, in poultry farms, polystyrene is used in water suppliers, feeding stations, or in the containers where living broilers are transported to the slaughterhouses. In food packaging, polystyrene is commonly used in chicken and beef packaging or in fruit packaging [25]. The results of this study showed that in both polystyrene and stainless steel, the mean OD_630_ observed was significantly higher with the 1/20 TSB growth medium in comparison with the other growth media tested. Therefore, it is possible that the results of biofilm assays performed with 1/20 TSB enhanced the biofilm formation of *Salmonella* strains, and their capacity to produce biofilm on the presence of food residues is lower in comparison with 1/20 TSB. In contrast with these results, a research carried out by Li et al. [14] observed that *Salmonella* strains formed more biofilm with meat juice than with the common laboratory growth medium Mueller-Hinton (MH) at 37 °C in polystyrene and glass surfaces. These different results could be due to the conditions used in both studies. It has been observed that high nutrient concentration media, such as MH or TSB without dilution, combined with temperatures of 37 °C results in lower biofilm formation by *Salmonella* strains [10,26].

Related to this, the temperature is another factor that influences biofilm formation. With the exception of TJ, the mean OD_630_ was higher at 22 °C than at 6 °C in the growth media used in this study. Lamas et al. [10] observed that 20 °C was the temperature at which *Salmonella* strains produced more biofilm with the 1/20 TSB growth medium. The same results were observed in this study for the 1/20 Milk and CMJ growth media. However, there were no significant differences in TJ between 6 °C and 22 °C. In this sense, Koukkidis et al. [27] observed that salad juices highly influenced growth at refrigerated temperatures, motility, and biofilm formation in *Salmonella*. Therefore, it is possible that other factors influence biofilm formation with these growth media. It is remarkable that, with one exception, all the strains were able to produce biofilm on polystyrene and stainless steel surfaces with all the growth media tested in this study. Both polystyrene and stainless steel surfaces are commonly found in the different steps of the food chain. The combination of food residues and *Salmonella* cells in these surfaces could result in biofilm formation and therefore cross-contamination of food products in contact with these surfaces with *Salmonella*. These results are a major of concern in public health and reflect the importance of maintaining adequate hygiene and disinfection methods to avoid the presence of food-borne pathogens biofilms, both in domestic refrigerators and storage chillers.

Raw milk is characterized for its complex microbial community composed of a wide range of bacterial genera that are able to form biofilm [28,29]. Although the influence of milk in biofilm formation has been evaluated in food-borne pathogens, such as *Listeria monocytogenes* [29] or *Staphylococcus aureus* [30], and spoilage bacteria [31], to the best of our knowledge the influence of milk in *Salmonella* biofilm formation has still not been evaluated. In this study, all the strains tested formed biofilm on stainless steel and polystyrene at 6 °C and 22 °C with 1/20 milk. Also, in polystyrene at 22 °C, the mean OD_630_ average was significantly higher in 1/20 milk than in other food residue media. It is also remarkable that the OD_630_ values obtained for *S. enterica* subsp. *salamae* strains at 22 °C in polystyrene were three times higher for 1/20 TSB than for the growth media TJ and CMJ. Therefore, this study demonstrates that *Salmonella* strains are able to produce biofilm on the presence of milk in both temperatures and surfaces. These results highlight the importance of good hygiene and disinfection practices in dairy equipment such as bulk tanks. Quorum-sensing molecules can increase biofilm formation in *Salmonella*, and this cell mechanism could play an important role for bacterial communication in multispecies biofilms [32,33]. Due to the microbiota composition of raw milk, *Salmonella* cells can integrate in biofilms formed by other bacterial genera as a response to quorum-sensing molecules produced by other microorganisms and liberated to the milk.

The strains used in this study were isolated from poultry houses, and all these strains were able to produce biofilm on all the surfaces and temperatures tested in this study with the growth medium CMJ. This result indicates that meat juice is a nutrient source for *Salmonella* in the food processing environment, allowing their biofilm formation. Different to polystyrene, in the stainless steel surface the mean OD_630_ value was higher in CMJ assays than in TJ and 1/20 milk assays at 6 °C and 22 °C. Therefore, it is possible that some compounds of CMJ facilitate the *Salmonella* biofilm formation on this surface. In this sense, it has been proposed that chicken meat juice used in laboratory could present residual quorum-sensing molecules that enhance the biofilm formation in food-borne pathogens. Also, Li et al. [14] observed that aflagellated mutants of *Campylobacter* and *Salmonella* increased their biofilm-forming ability when surfaces were pre-coated with a meat juice layer. Thus, the particles of meat juice could promote the initial attachment of *Salmonella* cells to inert contact surfaces and allow biofilm formation.

*Salmonella* strains have been related with fruit, nut, or vegetable outbreaks [34,35,36]. In this sense, *Salmonella* contamination of vegetables can be originated by contaminated composed manure, soil, animals, or irrigation and wash water [37]. Therefore, the capacity of Salmonella strains from poultry houses to produce biofilms on the surface of vegetables is a major public health problem. Different studies have observed that *Salmonella* is able to produce biofilm on parsley, rocket leaves, lettuce cucumber, and tomatoes [38]. Koukkidis et al. [27] observed that salad leaf juices enhanced the motility and biofilm produced by *Salmonella*. In this study, tomato juice was used as one growth medium to evaluate the effect of this extract in the ability of *Salmonella* strains to produce biofilm on polystyrene and stainless steel. All the *Salmonella* strains tested in this study produced biofilm on stainless steel at 6 °C and 22 °C. However, in polystyrene, five strains did not produce biofilm at 22 °C and one strain did not produce biofilm at 6 °C. Therefore, TJ seems to favor biofilm formation in stainless steel more than in polystyrene. Also, it is interesting that there were no significant differences between 6 °C and 22 °C in biofilm formation. It is possible that some tomato compounds improve biofilm formation at low temperatures. In this sense, the previously mentioned study by Koukkidis et al. [27] also observed that salad leaf juices enhance *Salmonella* growth at refrigeration temperatures. In addition, food residues such as carrot can have a protective effect in *Salmonella* cells’ adherence to stainless steel [39]. The results of this study showed that *Salmonella* strains isolated from poultry houses produce biofilm on the presence of tomato residues.

## 5. Conclusions

*Salmonella* is one of the principal food-borne pathogens around the world. Authorities of different countries have adopted control strategies to reduce the prevalence of *Salmonella* in the food chain. However, the ability of *Salmonella* to produce biofilm is one of the main factors that make difficult their eradication from the food chain. Most of the studies carried out until now evaluated *Salmonella* biofilm formation using common laboratory growth media. However, biofilm formation is highly dependent on environmental conditions, and it is important to use food residues in biofilm assays to obtain results as close as possible to the real conditions of the food chain. The results of this study clearly showed that *Salmonella* strains isolated from poultry houses can produce biofilm both at 22 °C and 6 °C in stainless steel and polystyrene. Although biofilm formation was observed with all the growth media used, biofilm formation was significantly higher with the common laboratory growth medium. In this study, the laboratory growth medium 1/20 TSB was used, which enhances biofilm formation in *Salmonella*. This fact explains the high differences found between laboratory growth media and food residues. However, the effect of food residues in biofilm formation should not be underestimated. Future studies are necessary to confirm the results obtained in this study and to evaluate the effect of food residues in transcriptome of *Salmonella* cells. These data will allow the discovery of metabolic pathways involved in the interaction between *Salmonella* cells and food residues. Finally, it is necessary to develop standardized methods in biofilm assays to make possible the direct comparison of results obtained from different laboratories.

## Figures and Tables

**Table 1:** *Salmonella enterica* strains selected for this study and the morphotype produced under different incubation temperatures tested in this study (6 °C and 22 °C).

	Morphotype	
Serotype/subspecies	6 °C	22 °C
*S.* Typhimurium Lhica T1	SACW	RADR
*S.* Typhimurium Lhica T4	SACW	RADR
*S.* Typhimurium Lhica T5	SACW	RADR
*S.* Typhimurium Lhica T6	SACW	RADR
*S.* Enteritidis Lhica ET1	SACW	RADR
*S.* Bardo Lhica B2	SACW	RADR
*S.* Newport Lhica N5	SACW	RADR
*S.* Infantis Lhica I4	SACW	RADR
*S.* Infantis Lhica I5	SACW	RADR
*S. enterica* subsp. *arizonae* serovar 48:z4,z23,z32:-Lhica AZ2	SACW	SAW
*S. enterica* subsp. *arizonae* serovar 48:z4,z23:-Lhica AZ6	SACW	SAW
*S. enterica* subsp. *salamae* serovar 4,12:b:-Lhica SA3	SACW	RADR
*S. enterica* subsp. *salamae* serovar 6,8:g,m,t:-Lhica SA2	SACW	RADR

RDAR, red, dry, and rough; SAW, smooth and white; SACW, soft and completely white.

**Table 2:** Main motility (mm) of *Salmonella* strains tested in this study at 22 °C and 6 °C. Asterisks indicate significant differences (*p* < 0.05) in each strain between the two temperatures.

Strain	Motility (mm) at 22 °C	Motility (mm) at 6 °C
*S*. Typhimurium Lhica T1	15.50 ± 0.50	0.00 ± 0.00 *
*S*. Typhimurium Lhica T4	21.00 ± 1.00	6.00 ± 1.00 *
*S*. Typhimurium Lhica T5	25.10 ± 2.10	0.83 ± 0.29 *
*S*. Typhimurium Lhica T6	19.66 ± 1.53	2.17 ± 0.29 *
*S*. Enteritidis Lhica ET1	25.40 ± 3.20	2.00 ± 1.00 *
*S*. Bardo Lhica B2	19.83 ± 1.26	1.33 ± 0.58 *
*S*. Newport Lhica N5	19.50 ± 1.50	0.00 ± 0.00 *
*S*. Infantis Lhica I4	15.50 ± 0.50	6.00 ± 0.50 *
*S*. Infantis Lhica I5	12.00 ± 1.00	1.23 ± 0.25 *
*S*. *enterica* subsp. *arizonae* serovar 48:z4,z23,z32:-Lhica AZ2	20.07 ± 0.51	0.73 ± 0.40 *
*S*. *enterica* subsp. *arizonae* serovar 48:z4,z23:-Lhica AZ6	19.90 ± 0.79	1.00 ± 0.50 *
*S*. *enterica* subsp. *salamae* serovar 4,12:b:-Lhica SA3	15.33 ± 0.76	0.83 ± 0.57 *
*S*. *enterica* subsp. *salamae* serovar 6,8:g,m,t:-Lhica SA2	15.17 ± 1.04	1.83 ± 0.29 *
Average	18.62 ± 3.97	1.77 ± 1.96 *

**Table 3:** Biofilm formation on polystyrene plates with the four different growth media used at 6 °C and 22 °C by *Salmonella* strains tested in this study.

Strains	Media							
	1/20 TSB		1/20 Milk		TJ		CMJ	
St.	6 °C	22 °C	6 °C	22 °C	6 °C	22 °C	6 °C	22 °C
T1	0.112 ± 0.007 ^a^	0.366 ± 0.089 ^a,^*	0.092 ± 0.010 ^a,b^	0.262 ± 0.077 ^b,^*	0.079 ± 0.002 ^b^	0.061 ± 0.004 ^d^	0.083 ± 0.003 ^b^	0.100 ± 0.015 ^c^
T4	0.149 ± 0.012 ^a^	0.395 ± 0.159 ^a,^*	0.084 ± 0.011 ^b^	0.139 ± 0.036 ^b,^*	0.083 ± 0.007 ^b^	0.059 ± 0.006 ^d,^*	0.080 ± 0.004 ^b^	0.088 ± 0.016 ^c^
T5	0.146 ± 0.045 ^a^	0.169 ± 0.057 ^a^	0.099 ± 0.026 ^b^	0.160 ± 0.089 ^a^	0.078 ± 0.009 ^c^	0.080 ± 0.005 ^c^	0.077 ± 0.005 ^c^	0.111 ± 0.013 ^b,^*
T6	0.152 ± 0.012 ^a^	0.219 ± 0.038 ^a,^*	0.090 ± 0.009 ^b^	0.144 ± 0.052 ^b,^*	0.057 ± 0.005 ^d^	0.071 ± 0.010 ^d^	0.076 ± 0.004 ^c^	0.113 ± 0.018 ^c,^*
ET1	0.112 ± 0.007 ^a^	0.461 ± 0.069 ^a,^*	0.101 ± 0.014 ^a,b^	0.199 ± 0.032 ^b,^*	0.082 ± 0.008 ^b,c^	0.088 ± 0.010 ^c^	0.079 ± 0.010 ^c^	0.091 ± 0.015 ^c,^*
B2	0.232 ± 0.037 ^a^	0.452 ± 0.042 ^a,^*	0.108 ± 0.026 ^b^	0.160 ± 0.033 ^b,^*	0.080 ± 0.010 ^c^	0.116 ± 0.012 ^c,^*	0.085 ± 0.003 ^c^	0.156 ± 0.022 ^b^
N5	0.201 ± 0.022 ^a^	0.424 ± 0.049 ^a,^*	0.105 ± 0.017 ^b^	0.191 ± 0.035 ^b,^*	0.110 ± 0.013 ^b^	0.081 ± 0.002 ^c^	0.097 ± 0.025 ^b^	0.105 ± 0.034 ^c^
I4	0.076 ± 0.008 ^a^	0.131 ± 0.025 ^a,^*	0.104 ± 0.039 ^a^	0.117 ± 0.061 ^a^	0.100 ± 0.021 ^a^	0.118 ± 0.032 ^a^	0.111 ± 0.039 ^a^	0.116 ± 0.025 ^a^
I5	0.158 ± 0.012 ^a^	0.404 ± 0.135 ^a,^*	0.104 ± 0.021 ^b^	0.146 ± 0.021 ^b^	0.082 ± 0.003 ^c^	0.125 ± 0.022 ^b,^*	0.079 ± 0.004 ^c^	0.085 ± 0.022 ^c^
AZ3	0.115 ± 0.013 ^a^	0.142 ± 0.063 ^a^	0.080 ± 0.010 ^b^	0.089 ± 0.020 ^b^	0.083 ± 0.005 ^b^	0.060 ± 0.009 ^c,^*	0.079 ± 0.006 ^b^	0.089 ± 0.019 ^b^
AZ6	0.131 ± 0.015 ^a^	0.098 ± 0.011 ^a,^*	0.087 ± 0.020 ^b^	0.100 ± 0.018 ^a^	0.090 ± 0.010 ^b^	0.067 ± 0.010 ^b,^*	0.082 ± 0.007 ^c^	0.097 ± 0.012 ^a^
SA2	0.642 ± 0.098 ^a^	0.563 ± 0.034 ^a^	0.130 ± 0.040 ^b^	0.300 ± 0.060 ^b,^*	0.079 ± 0.010 ^c^	0.081 ± 0.011 ^d^	0.080 ± 0.020 ^c^	0.121 ± 0.022 ^c,^*
SA3	0.433 ± 0.019 ^a^	0.566 ± 0.061 ^a,^*	0.124 ± 0.032 ^b^	0.314 ± 0.039 ^b,^*	0.081 ± 0.001 ^c^	0.066 ± 0.009 ^d,^*	0.077 ± 0.012 ^c^	0.096 ± 0.009 ^c,^*
$\bar{X}$	0.210 ± 0.161 ^a^	0.341 ± 0.165 ^a,^*	0.101 ± 0.020 ^b^	0.179 ± 0.070 ^b,^*	0.084 ± 0.015 ^b^	0.083 ± 0.023 ^c^	0.081 ± 0.014 ^b^	0.105 ± 0.022 ^c,^*

Different letters in the same row and the same temperature indicate significant differences (*p* < 0.05). Asterisks indicate significant differences (*p* < 0.05) between the two temperatures for the same growth medium. Strains code corresponds to those in Table 1. St.: strains; $\bar{X}$: Average. Tryptic Soy Broth 1/20 at 1/20 (*w*/*w*) (1/20 TSB); Milk at 1/20 (*w*/*w*) (1/20 Milk); Tomato Juice (TJ); Chicken meat juice (CMJ).

**Table 4:** Biofilm formation on stainless steel coupons with the four different growth media used at 6°C and 22°C by *Salmonella* strains tested in this study.

Strains	Media							
	1/20 TSB		1/20 Milk		TJ		CMJ	
St.	6 °C	22 °C	6 °C	22 °C	6 °C	22 °C	6 °C	22 °C
T1	0.161 ± 0.011 ^a^	0.212 ± 0.010 ^a,^*	0.098 ± 0.010 ^c^	0.118 ± 0.008 ^b^	0.090 ± 0.015 ^c^	0.080 ± 0.010 ^c^	0.123 ± 0.011 ^b^	0.127 ± 0.012 ^b^
T4	0.182 ± 0.009 ^a^	0.228 ± 0.010 ^a,^*	0.080 ± 0.008 ^c^	0.107 ± 0.012 ^b^	0.083 ± 0.006 ^c^	0.111 ± 0.013 ^b^	0.131 ± 0.005 ^b^	0.112 ± 0.011 ^b^
T5	0.134 ± 0.010 ^a^	0.172 ± 0.010 ^a,^*	0.076 ± 0.007 ^b^	0.106 ± 0.012 ^b,^*	0.073 ± 0.015 ^b^	0.079 ± 0.012 ^c^	0.071 ± 0.028 ^b^	0.101 ± 0.013 ^b,^*
T6	0.124 ± 0.007 ^a^	0.124 ± 0.007 ^a,b^	0.089 ± 0.010 ^b^	0.111 ± 0.009 ^b,^*	0.060 ± 0.004 ^c^	0.056 ± 0.011 ^c^	0.120 ± 0.014 ^a^	0.136 ± 0.020 ^a^
ET1	0.199 ± 0.010 ^a^	0.247 ± 0.008 ^a,^*	0.077 ± 0.010 ^b^	0.109 ± 0.008 ^b,^*	0.081 ± 0.009 ^b^	0.069 ± 0.009 ^c^	0.083 ± 0.019 ^b^	0.107 ± 0.009 ^b^
B2	0.141 ± 0.012 ^a^	0.182 ± 0.008 ^a,^*	0.100 ± 0.013 ^b^	0.121 ± 0.009 ^b,^*	0.059 ± 0.010 ^c^	0.079 ± 0.010 ^c^	0.104 ± 0.009 ^b^	0.134 ± 0.011 ^b,^*
N5	0.122 ± 0.011 ^a^	0.162 ± 0.009 ^a,^*	0.090 ± 0.009 ^b^	0.122 ± 0.008 ^b,^*	0.087 ± 0.010 ^b^	0.089 ± 0.012 ^c^	0.139 ± 0.021 ^a^	0.150 ± 0.009 ^a^
I4	0.080 ± 0.012 ^a^	0.130 ± 0.011 ^a,^*	0.082 ± 0.010 ^a^	0.110 ± 0.008 ^a,^*	0.060 ± 0.008 ^b^	0.067 ± 0.005 ^b^	0.085 ± 0.010 ^a^	0.119 ± 0.010 ^a,^*
I5	0.178 ± 0.002 ^a^	0.218 ± 0.012 ^a,^*	0.080 ± 0.010 ^c^	0.104 ± 0.008 ^c,^*	0.078 ± 0.012 ^c^	0.055 ± 0.015 ^d,^*	0.119 ± 0.012 ^b^	0.139 ± 0.009 ^b,^*
AZ3	0.099 ± 0.011 ^a^	0.121 ± 0.009 ^a,^*	0.101 ± 0.021 ^a^	0.111 ± 0.010 ^a^	0.092 ± 0.022 ^a^	0.069 ± 0.012 ^b^	0.099 ± 0.010 ^a^	0.119 ± 0.005 ^a,^*
AZ6	0.098 ± 0.006 ^a^	0.131 ± 0.010 ^a,^*	0.099 ± 0.018 ^a^	0.101 ± 0.008 ^b^	0.100 ± 0.020 ^a^	0.070 ± 0.005 ^c,^*	0.102 ± 0.030 ^a^	0.139 ± 0.026 ^a,^*
SA2	0.155 ± 0.015 ^a^	0.198 ± 0.023 ^a,^*	0.103 ± 0.014 ^b^	0.110 ± 0.010 ^b,c^	0.110 ± 0.020 ^b^	0.098 ± 0.011 ^c^	0.108 ± 0.020 ^b^	0.135 ± 0.011 ^b,^*
SA3	0.159 ± 0.012 ^a^	0.188 ± 0.010 ^a,^*	0.100 ± 0.006 ^b^	0.115 ± 0.009 ^b^	0.099 ± 0.010 ^b^	0.120 ± 0.010 ^b^	0.104 ± 0.016 ^b^	0.129 ± 0.013 ^b^
$\bar{X}$	0.141 ± 0.036 ^a^	0.178 ± 0.042 ^a,^*	0.090 ± 0.010 ^b^	0.111 ± 0.012 ^b,c,^*	0.088 ± 0.016 ^b^	0.084 ± 0.020 ^c^	0.107 ± 0.035 ^b^	0.127 ± 0.014 ^b^*

Different letters in the same row and the same temperature indicate significant differences (*p* < 0.05). Asterisks indicate significant differences (*p* < 0.05) between the two temperatures for the same growth medium. Strains code corresponds to those in Table 1. St.: strains; $\bar{X}$: Average.
